# No Evidence for Prolonged Visible Persistence in Patients with Schizophrenia

**DOI:** 10.1371/journal.pone.0058940

**Published:** 2013-03-11

**Authors:** Cathleen Grimsen, Andreas Brand, Manfred Fahle

**Affiliations:** 1 Department of Human Neurobiology, University of Bremen, Bremen, Germany; 2 Klinikum Bremen-Ost, Bremen, Germany; 3 Department of Human Neurobiology, University of Bremen, Bremen, Germany; 4 The Henry Wellcome Laboratories for Vision Sciences, City University London, London, United Kingdom; Baylor College of Medicine, United States of America

## Abstract

**Background:**

Temporal visual processing is strongly deteriorated in patients with schizophrenia. For example, the interval required between a visual stimulus and a subsequent mask has to be much longer in schizophrenic patients than in healthy controls. We investigated whether this deficit in temporal resolution is accompanied by prolonged visual persistence and/or deficient temporal precision (temporal asynchrony perception).

**Methodology/Principal Findings:**

We investigated visual persistence in three experiments. In the first, measuring temporal processing by so-called backward masking, prolonged visible persistence is supposed to decrease performance. In the second experiment, requiring temporal integration, prolonged persistence is supposed to improve performance. In the third experiment, we investigated asynchrony detection, as another measure of temporal resolution. Eighteen patients with schizophrenia and 15 healthy controls participated. Asynchrony detection was intact in the patients. However, patients' performance was inferior compared to healthy controls in the first two experiments. Hence, temporal processing in schizophrenic patients is indeed significantly impaired but this impairment is not caused by prolonged temporal integration.

**Conclusions/Significance:**

Our results argue against a generally prolonged visual persistence in patients with schizophrenia. Together with the preserved ability of patients, to detect temporal asynchronies in permanently presented stimuli, the results indicate a more specific deficit in temporal processing of schizophrenic patients.

## Introduction

Patients with schizophrenia show deficits in many domains including perceptual, cognitive and executive abilities. Deficits of early visual information processing are of particular interest because understanding perceptual dysfunctions will add important insights into the psychopathology of schizophrenia and might help to explain disturbed higher cognitive and social abilities [1]–[4]. One focus of research in schizophrenic patients is on processing of briefly presented visual stimuli usually followed by a mask [3]. Most often used are visual *backward masking* techniques where a briefly presented target is followed by a (spatially overlapping) masking stimulus. Target and mask are separated in time by a variable stimulus onset, called SOA (stimulus onset asynchrony). A large number of studies show that schizophrenic patients are impaired in backward masking, that is they need significant longer SOA's to reach the same performance level in target detection or discrimination as healthy controls do [3], [5]–[15]. In addition to the backward masking deficits there exist deficits in so-called two-flash fusion paradigms, where the task of the observer is not to detect or to identify the first of two successive images (like in backward masking), but to indicate whether or not the two (spatially overlapping) images are perceived as separate entities, i.e. whether the blank screen between them is clearly visible. Most studies found increased SOA's for schizophrenic patients in this task [16]–[18], but also lack of an effect has been reported [19]. Schizophrenic patients are also impaired in temporal simultaneity judgment, a task very similar to the two flash fusion paradigm. Here, the images presented are not spatially overlapping, and patients need longer time intervals to perceive two discrete events as “one-after-the-other”. This effect is stronger for bimodal stimuli [20] or when a visual prime is additionally presented [21]. Both studies concluded that schizophrenic patients have an extended window of simultaneity.

To explain the visual deficits in schizophrenic patients, it was often proposed that information processing is prolonged [22], [23] and that the disturbances must be based on an early-stage processing deficit [3], [24]. The underlying theory was originally proposed by Breitmeyer and Ganz (1976) who explained the phenomenon of visual masking through the interplay of magnocellular and parvocellular channels. The magnocellular channel responds in a transient manner preferentially to stimuli with low-spatial and high-temporal frequency, whereas the parvocellular channels' response is sustained and prefers processing of high-spatial and low-temporal frequency patterns [25]. In the case of backward masking the difference between both channels in response latencies and neural persistence may result in an interference of processing between the first stimulus (target) and the second stimulus (mask) either by interruption or integration [26]. Many authors attribute the backward masking deficit in schizophrenic patients to an aberrant magnocellular function, highlighting the high temporal resolution of that processing channel [14], [27]–[30]. One implication of these theories would be that visible persistence is extended in schizophrenic patients. The term visual persistence refers to the time during which the response to a brief visual stimulus continues although its physical presentation has ended [31]. A stimulus representation persisting in the nervous system after the end of stimulus presentation may be fused with the mask and therefore be masked, hence the stimulus cannot be identified [2], [31]–[35]. This mechanism of prolonged integration would explain the need of longer SOAs in schizophrenic patients because of prolonged persistence. Slaghuis [35] provided further evidence for increased visible persistence as the cause for longer SOÁs in backward masking. He found that schizophrenic patients need longer integration times to reach a contrast threshold. These longer integration times correspond to a longer visible persistence, hence patients are more susceptible to backward masking. Patients with negative symptoms showed a significant correlation between lower contrast thresholds and longer masking SOA's.

All paradigms described above investigated the temporal domain of visual information processing by means of briefly and consecutively presented stimuli, where the task is either to temporally separate or else temporally integrate two stimuli (backward masking versus two-flash-fusion) or to assess the temporal asynchrony (simultaneity). In our study we combined three paradigms to analyze aspects of possible (dys)function of visual temporal processing in patients with schizophrenia.

**Figure 1:**
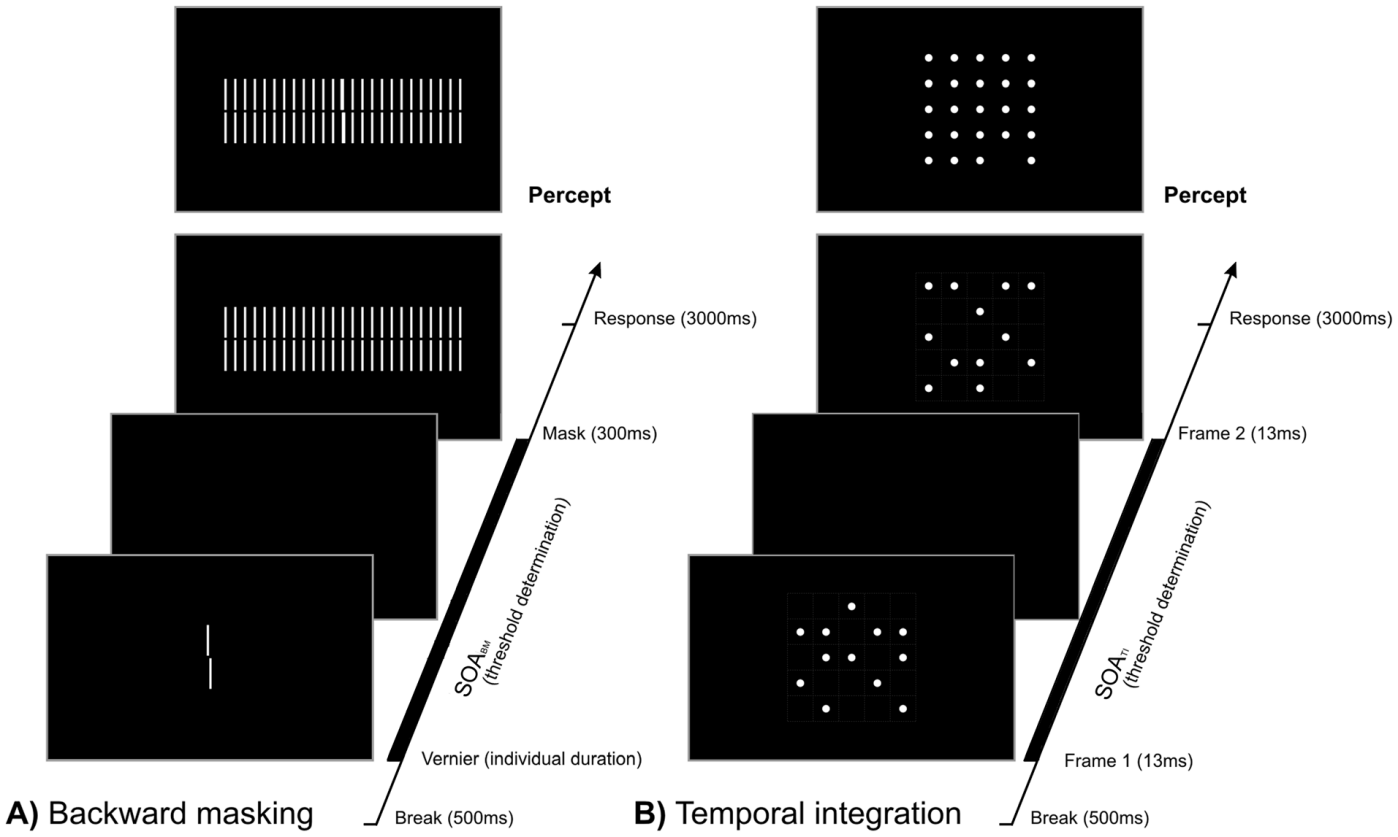
Schematic illustration of experimental setups. **A**) The shine-through masking paradigm. A vernier, i.e. two vertical bars that are slightly offset in the horizontal direction, is followed by a grating after a variable SOA, i.e. a blank screen. The task of the observers is to indicate the offset direction of the vernier which is either offset to the left or right. The following grating makes this task difficult, particularly for short SOAs. **B**) The temporal integration paradigm. Two frames with 12 dots each are presented with a variable SOA (the grid is only shown for illustrative purpose and was not present in the experiment). The task of the observer was to indicate the side of the missing dot. The task becomes more difficult with longer SOAs.

**Figure 2:**
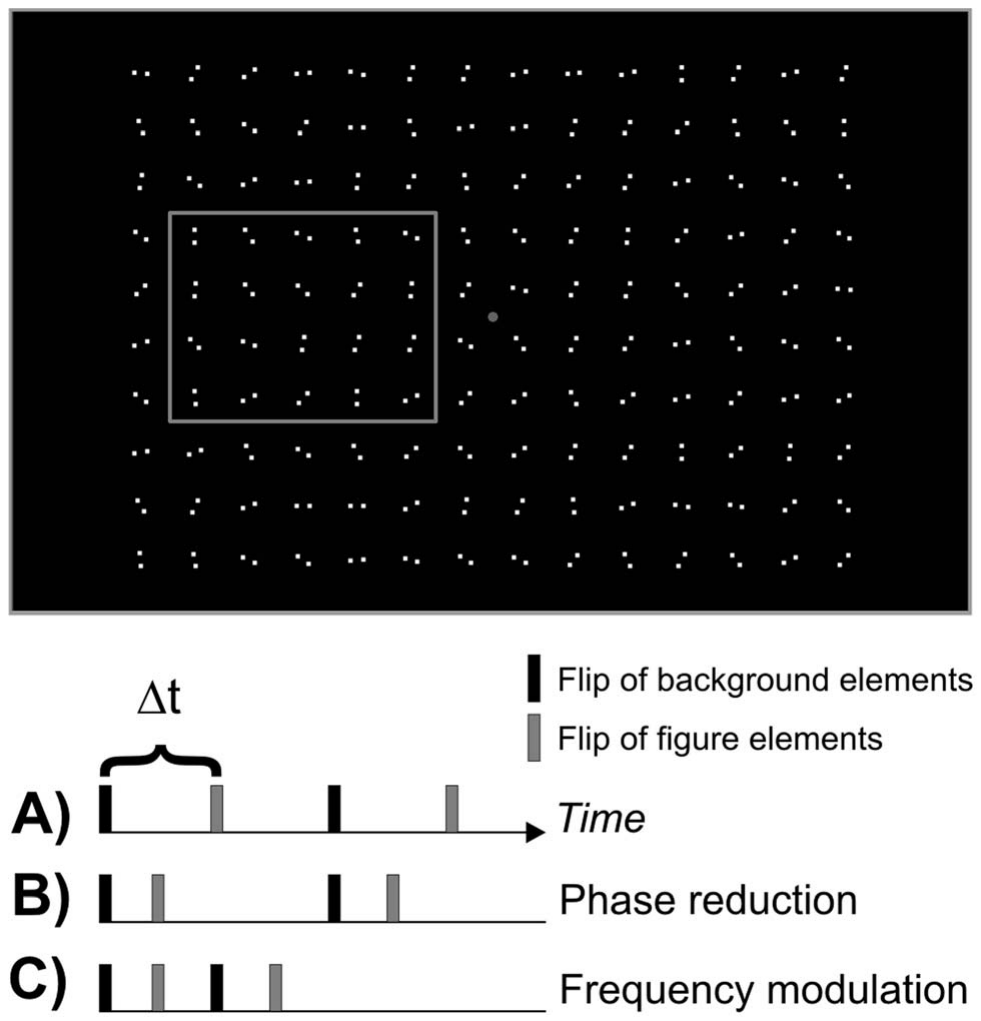
Schematic illustration of temporal figure-ground-segmentation. The task of the observer was to indicate the side of the rectangle. The grey accentuation of the rectangle is only for illustrational purposes and was not displayed during the experiment. (A) In the beginning of the measurement there is a fixed difference (Δt) between target and background flips, defining also the frequency of flips. By reducing Δt either while (B) keeping the basis frequency constant (*phase reduction*) or by (C) reducing Δt and therefore increasing the frequency (*frequency modulation*), the task becomes gradually more difficult.

First, we used a stimulus introduced by Herzog et al., for which a strong backward masking effect in schizophrenic patients has been repeatedly demonstrated [36–39,40, see figure 1]. To test temporal integration we used the method of integrating form parts [31] by modifying a task from Hogben and DiLollo [41]. In this task observers have to detect a missing dot in an array of dots [41, see figure 1B]. This task allows the direct measurement of visual persistence within a forced choice task, because solving the task demands the superimposition of two visual parts separated in time to obtain an interpretable percept (see Methods). If visual persistence is prolonged in schizophrenic patients an extended stimulus-onset asynchrony between the two parts should be possible, i.e. patient's performances should be better than the ones of controls. Finally, to test temporal (a)synchrony perception we used a figure-ground segmentation task which relies on purely temporal cues [42]. To solve this task observers have to detect differences in temporal asynchrony between target and background elements (figure 2). The stimulus we used does not contain motion cues that would allow solving the task. If there is an extended window of simultaneity in schizophrenic patients they should have elevated perceptual thresholds in this task.

Examining these three complementary paradigms of temporal visual information processing in the same group of patients provides the basis to further analyze visual temporal dysfunction in schizophrenic patients.

## Materials and Methods

### Ethics Statement

The study was performed in accordance with the Declaration of Helsinki [43]. All observers were informed about the general purpose of the experiment and gave written consent. Subjects were told that they could quit the experiment at any time they wished. The study was approved by the ethics committee of the University of Bremen.

Three experiments were conducted. For the masking experiment, we used the well established shine-through masking paradigm for which patients with schizophrenia show strongly deteriorated performance [37], [38], [44]. In the second experiment, the same observers underwent a temporal integration task pioneered by Hogben and Di Lollo [41]. In the last experiment, we tested figure-ground segmentation by purely temporal cues using two different stimulus manipulations [42].

### Participants

Twenty-two patients with schizophrenia initially participated in the study. They were in-patients from the Centre for Psychiatry and Psychotherapy at the Klinikum Bremen-Ost. Diagnosis was made according to DSM-IV based on a clinical interview, the medical record, and interviews with the hospital staff. Exclusion criteria were age older than 50 years, diagnosis of a neurological disease including head trauma, and substance abuse during four weeks preceding the testings. Control subjects were screened for axis I disorders and a family history of psychiatric disorders. To ensure voluntary participation, patients with schizophrenia did not receive any financial nor any other compensation. Control subjects got a small financial benefit (12€), this was done mainly to compensate for the journey they had to incur. Four patients with too long vernier durations had to be excluded (see procedure). The demographical and clinical data of the remaining 18 patients and 15 controls are listed in table 1.

**Table 1:** Demographical, clinical and neurocognitive data of patients and controls.

	Schizophrenic patients		Healthy controls		t	p
N	18		15			
Male/Female	14/4		12/3			
	Mean	S.D.	Mean	S.D.		
Age (years)	33.2	7.6	26.8	4.6	2.9	**<.01**
Education (years)	13.0	3.5	13.8	2.1	0.5	n.s.
Visual acuity	1.4	0.3	1.5	0.2	-0.6	n.s.
Vernier duration (ms)	22.2	21.6*	16.0	5.1	0.3	n.s.
Duration of illness(years)	7.9	6.4				
CPZ (mg)	849.6	398.4				
SANS	8.4	3.8				
SAPS	3.0	3.7				
d2	44.2	9.3	65.3	12.4		**<.001**
MWT	55.5	8.9	63.1	10.7		**<.05**
LPS 3	55.1	6.4	60.6	6.9		**<.05**

S.D. = standard deviation, CPZ = chlorpromazin equivalents, SANS = Scale for the Assessment of Negative Symptoms, SAPS = Scale for the Assessment of Positive Symptoms, d2 = ‘Aufmerksamkeits-Belastungstest’, MWT = ‘Mehrfachwahl-Wortschatz-Test’, LPS = ‘Leistungspruefsystem’. *The high standard deviation is due to the fact that one patient had an individual vernier duration of 100 ms, whereas all others did not exceed 40 ms.

For the assessment of the psychopathological condition, the SANS and the SAPS were used [45], [46]. Diagnosis and psychopathological ratings were carried out by an experienced senior psychiatrist (A.B.) within the week following the day of testing. Psychopathological rating was based on the symptoms existing in the week preceding the rating. With this method we determined symptoms with a close temporal relation to the testing.

All patients were receiving neuroleptic medication: olanzapine, clozapine, risperidone, quetiapine, amisulpride, fluphenazine or promethazine. Two patients received two of these neuroleptics, one patient three of them. Two patients received lorazepam and one patient additionally zopiclone. Chlorpromazine equivalents were calculated according to the Agency for Healthcare Research and Quality [47].

### General set-up

Stimuli were displayed on an EIZO monitor F563-T controlled by a PC. Subjects observed the stimuli from a distance of 2.5 m in a room illuminated dimly by a background light (around 0.5 lx). A pixel comprised about 23’’ (arcsec, 60’’  =  1arcmin) at this distance. Stimuli were white on a dark background. Luminance of stimuli was approximately 80cd/m^2^. Background luminance was below 1 cd/m^2^ and, hence, Michelson contrast [(L*_max_*−L*_min_*)/(L*_max_*+L*_min_*)] was close to 1.0. Refresh rate was 100 Hz with a spatial resolution of 1024×768 pixels in the backward masking experiment and of 150 Hz with 640×480 pixels in the temporal integration and figure-ground segmentation tasks.

### Procedure

Initially we determined binocular visual acuity of patients and healthy controls by means of the Freiburg visual acuity test [48]. To participate in the following experiments, observers had to reach at least visual acuity of 0.8 (equivalent to 20/25 Snellen).

In all experiments participants gave their responses with two buttons for the required left-right decision, the button in the left hand was always used for “left” and the one in the right hand for “right”.

### Stimulus onset asynchrony in backward masking (SOA_BM_) for measuring temporal resolution

For the visual backward masking we used a vernier stimulus. The vernier stimulus was composed of two vertical bars that were slightly displaced in the horizontal direction (see Fig. 1). In each trial, the vernier offset was chosen randomly to be either to the right or left. Observers had to indicate the offset direction of the lower part of the vernier after each presentation (left/right; binary forced choice). Errors and omissions were followed by an auditory signal.

Before the backward masking experiment, we determined the critical vernier duration for each observer by an adaptive staircase procedure. The individual result of this pre-test was used for the backward masking experiment, guaranteeing that task difficulty was comparable for all observers. The pretest was also used to familiarize participants with the task. We presented a single vernier with a constant horizontal offset of 46’’ (3600”  = 60arcmin  =  1arcdeg). For each observer, we tried to find the vernier duration for which performance for offset discrimination was about 75% correct (threshold). Presentation duration of verniers started at 150 ms and was subsequently adjusted by the adaptive procedure PEST [49]. To join the following masking experiment, a vernier duration shorter than 100 ms was required, to enable masking effects (it is difficult to mask stimuli which are presented longer than 100ms). Minimal vernier duration was 10 ms due to the refresh rate of the monitor of 100 Hz. Four patients did not meet this predefined criterion and were excluded from the study.

In the actual backward masking experiment the vernier target was followed by a grating mask. We determined the critical time interval for the identification of this horizontal vernier offset in visual backward masking (SOA_BM_). The mask was a grating comprising 25 elements (see Figure 1A). The grating elements were aligned verniers, i.e., verniers without horizontal offset. Vernier and grating elements were 21 arcmin long, separated by a small vertical gap of 50 arcsec. The horizontal distance between elements of the grating was 3.3 arcmin. The vernier and the central element of the grating appeared always in the middle of the screen. The (virtual) middle of the vernier was in the centre of the screen. Masking gratings appeared for 300 ms. We adjusted the vernier-mask inter stimulus interval (SOA_BM_) to yield a performance level of 75% correct responses for a vernier with a constant offset of 70”. The SOA_BM_ is defined as the time difference between disappearance of the vernier and mask onset (Figure 1A) and was determined by the adaptive procedure PEST [49].

The task for the observer was to indicate the offset direction of the vernier by appropriate button press. Auditory feedback (beep) was provided in case of errors and omissions. Two thresholds were determined in blocks of 80 presentations each. The mean of both SOA_BM_ thresholds served for statistical analysis.

### Stimulus onset asynchrony in Temporal Integration (SOA_TI_) for coherent stimulus perception

In the temporal integration paradigm, two stimuli containing 12 dots each (with diameter 1.9 arcmin) were displayed for 13.3 ms each, separated by a variable inter stimulus interval (SOA_TI_). Together the 2×12 dots produced a 5×5 square with one dot missing, either on the left or on the right side of the stimulus (none of the five dots at the vertical midline was ever omitted, see Figure 1 B). All dots were located on a virtual matrix, with horizontal and vertical separations of 20 arcmin. The task of the observers was to indicate on which side (left or right) a dot was missing by button press.

We estimated the threshold (performance level corresponding to 75% correct) by means of an adaptive staircase procedure, QUEST [50]. The staircase started with an SOA_TI_ of 0ms and was completed after 80 trials. The measurement was repeated once and the mean served for statistical analysis.

### Critical Time Interval in Temporal Figure-Ground-Segmentation for temporal precision

Finally, we conducted two experiments testing asynchrony detection. We used a paradigm introduced by Kandil and Fahle [42] presenting 14 (width) ×10 (height) colons in random orientation, which rotated around their imaginary midpoint by 90° at defined points in time (see Figure 2). Individual dots had a diameter 1.2 arcmin and the distance between the two dots of a colon was about 6.2 arcmin. Colons were separated by 25’ both in horizontal and vertical direction. The target was a rectangle either to the left or right of the fixation point generated by 4×5 colons that flipped at a point in time differing from that of the background colons (Figure 2). Background and target elements flipped at different times, and these time differences were varied in two different ways. In the *phase reduction* condition, target and background elements flipped with a constant frequency of 5 Hz during the whole presentation. We determined the threshold delay between background and target flip for which observers could identify the side of appearance of the rectangle. Due to the basic frequency of 5 Hz (one flip every 200 ms for target and one flip every 200 ms for background elements) the adaptive staircase started with a value of 100 ms, because this is the maximal time difference between target and background flips. As the frequency stays constant the staircase modulated only the time difference between target and background flips.

In the *frequency modulation* condition, the delay between subsequent target flips was gradually reduced by increasing the frequency, starting at a frequency of ∼3.8 Hz (one flip every 266 ms for target and one flip every 266 ms for background elements). This corresponds to a delay of 133 ms as a start value for the adaptive staircase procedure (see figure 2). The delay between target and background flips was always maximal for the frequency chosen. So by increasing the frequency for example to 5 Hz, the delay between target and background flips becomes shorter (in this case 100 ms) and the task more difficult. The threshold in this task is the minimal delay between target and background flips. For both conditions the stimulus was displayed for 3000 ms at maximum.

### Neuropsychological tests

In order to characterize our sample in more detail, we assessed sustained attention by means of the d2 test [51], global cognitive performance using the subtest 3 of the Horn “Leistungspruefsystem” LPS [52], and pre-morbid intelligence by a word recognition test “Mehrfachwahl-Wortschatz-Intelligenztest” MWT [53]. The tests were usually performed after the experimental session but at the latest three days afterwards.

### Statistical analysis

The data were analyzed by means of a general linear model for repeated measurements with the within-subject factor *SOA-condition* (backward masking vs. temporal integration) and the between-subjects factor *group* (patients vs. controls) with age as covariate. The same analysis was carried out for the figure-ground-segmentation experiment, but with the within-subject factor temporal threshold condition (phase reduction vs. frequency modulation). Post hoc comparisons were carried out by student t- tests for paired samples and univariate ANOVAS with age as covariate for comparison between groups. Level of significance was 0.05 in all tests.

## Results

### Psychopathology and Neuropsychological Tests

Demographical data, psychopathological data (SANS and SAPS), chlorpromazine equivalents and results of the neuropsychological tests are depicted in Table 1. Patients with schizophrenia revealed only weak psychotic symptoms (SAPS) and stronger negative symptoms (SANS). Patients performed substantially worse than healthy controls in all neurocognitive tests. There were no significant correlations between the measures of visual processing and SANS and SAPS, respectively.

### Temporal resolution in Backward Masking vs. Temporal Integration

**Figure 3:**
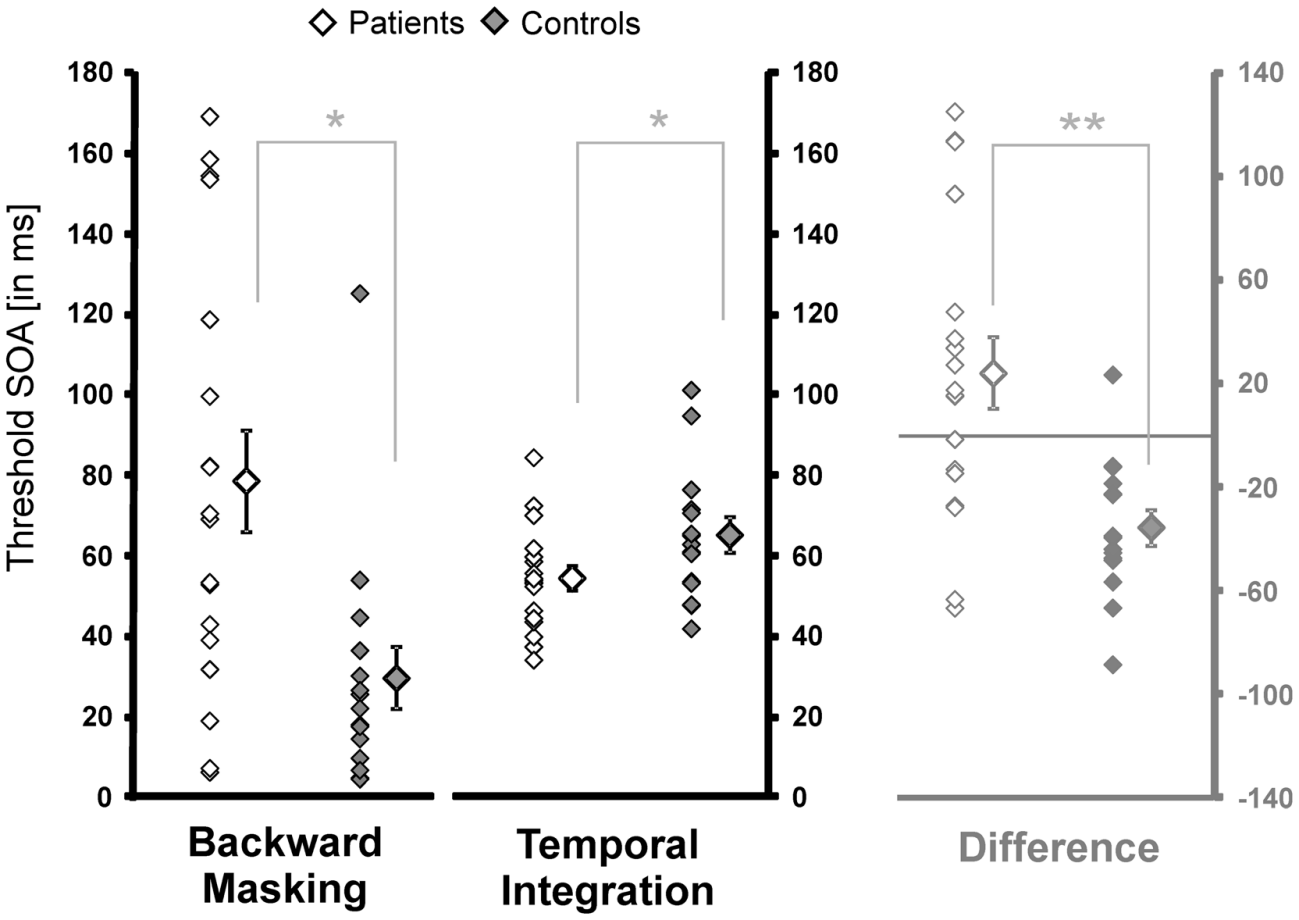
Results for backward masking and temporal integration. Individual thresholds and mean thresholds SOAs (±S.E.) for patients and controls are shown on the left. On the right the differences between the thresholds in the backward masking and the temporal integration task are shown to visualize the significant interaction. Values above zero indicate longer SOA_BM_ compared to SOA_TI_.

Results for patients and controls are shown in Figure 3. In the backward masking task mean threshold SOA for patients was 78.6 ms (±53.4 SD) and for controls 29.6 ms (±7.8 SD). In the temporal integration task mean threshold for patients was 54.4 ms (±3.0 SD) and for controls 65.1 ms (±4.3 SD). We analyzed the data using a repeated measurement ANOVA with factors condition (SOA_BM_ vs. SOA_TI_), group (patient vs. controls) and age as covariate. The main effects for condition [F_(1,30)_ = 1.15, p>.05] and group [F_(1,30)_ = 1.65, p>.05] were not significant, but there was a significant interaction [F_(1,30)_ = 8.00, p<.01]. To throw light on the interaction we computed the individual differences between SOA_BM_ and SOA_TI_. Patients mean was positive 24.2±57.9, whereas mean of controls was negative −35.6±57.9 (Figure 3).

Post hoc, we tested for group differences in each condition (backward masking and temporal integration) separately, using an univariate ANOVA with factor group and age as covariate. Here, a significant effect of group was found for both conditions (backward masking: F_(1,30)_ = 4.74, p<.05; temporal integration: F_(1,30)_ = 6.56, p<.05). Differences between thresholds within in each group both conditions were carried out using paired t-tests. Patients showed no significant difference between SOA_BM_ and SOA_TI_ (T_(17)_ = 1.8, p>.05) but controls showed a significant difference between both measurements (T_(14)_ = −5.2, p<.001).

### Temporal precision in Figure-Ground Segmentation

**Figure 4:**
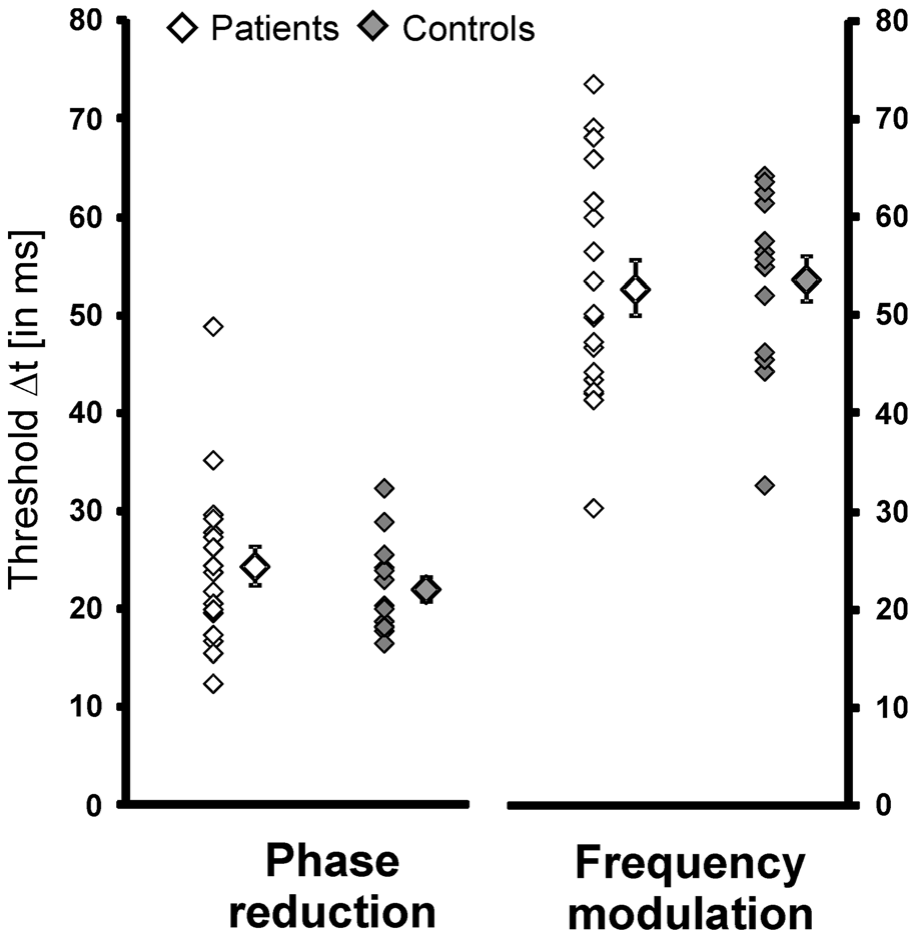
Results for temporal figure-ground-segmentation. Individual thresholds and mean thresholds (±S.E.) for phase reduction and frequency modulation are indicated.

Results for patients and controls are depicted in Figure 4. In the *phase reduction* condition patients needed on average a temporal delay of 24.3 ms (±8.4SD) and controls required 22.0 ms (±4.7 SD) to correctly detect the figure-ground difference in 75% of the trials. In the frequency *modulation condition,* thresholds of both groups were higher (patients: 52.7±11.8 ms; controls: 53.7±8.9). Analysis for repeated measurement with factors condition (phase vs. frequency) and group (patient vs. controls) as well as age as covariate showed a significant main effect for condition [F_(1,29)_ = 12.6, p<.01], but not for group [F_(1,29)_ =  .55, p = .46] and no interaction [F_(1,28)_ = 2.03, p = .17].

## Discussion

We tested the hypothesis that the much longer stimulus-onset-asynchronies in masking experiments required by patients with schizophrenia may be due to a reduced temporal resolution and/or increased temporal integration. In three separate experiments we compared three temporal aspects of sensory processing, namely: temporal resolution, integration and precision in a group of patients with schizophrenia, to compare the patients' results under different experimental conditions. In other words, we tested whether longer SOA's required by schizophrenic patients in a backward masking are accompanied by longer visible persistence and protracted simultaneity perception as determined through a temporal integration and a temporal figure-ground-segmentation task. The results are negative. Whereas schizophrenic patients need clearly longer SOA’s in the masking paradigm, SOA's were significantly shorter in the temporal integration paradigm and not the other way round as would be expected from a prolonged visible persistence (Fig. 3). Furthermore the significant interaction between groups and both tasks indicates that the critical time differnence (SOA) is declining from backward masking to temporal integration in schizophrenic patiens, while increasing in healthy controls.

Previous evidence for prolonged visible persistence comes from two flash fusion (TFF) or two pulse temporal resolution experiments where two identical stimuli (gratings) are presented at the same position, separated by a variable time interval. Schizophrenic patients need significantly longer intervals in this paradigm, so it was assumed that the first grating has a longer visible persistence [2], [18], [34]. Using letters instead of gratings Weiss et al. [19] failed to find prolonged visible persistence in a TFF task in schizophrenic patients. The different results reported may be explained by different experimental settings. The setup used in our study to determine visible persistence differs from those reported in the literature concerning the retinal position of the two flashes because relevant information was not spatially overlapping. This excludes the second frame from acting as backward mask and therefore does not interrupt the processing of the first frame. This design should achieve solving the task primarily with sustained channel activity. If visible persistence is primary due to sustained channel activity [31] the reduction supports the notion that there is also aberrant parvocellular function in schizophrenic patients.

As outlined in the introduction, previous studies have clearly demonstrated that temporal resolution of visual processing is strongly deteriorated in patients with schizophrenia. For example, a mask presented after a visual stimulus interferes with this stimulus over much longer time intervals in patients than in healthy controls [8], [9], [11], [14], [35], [54]. This strong effect was interpreted as indicating a general slowing down of neuronal processing as a consequence of schizophrenia. If this interpretation would be correct, it would constitute an important aspect of the psychopathology of schizophrenia. Slaghuis [35] showed that patients with negative symptoms have lower contrast sensitivity for a variety of gratings with different spatial and temporal frequencies. Furthermore these patients had longer target duration thresholds and stronger masking effects. They explained their findings in terms of slower visual processing at threshold and a reduction of transient channel responsiveness, resulting in extended temporal summation and longer visible persistence. Studies on the relation between contrast and masking as the one by Keri et al. [13] and studies supporting a prolonged visible persistence [35] used rather indirect measures to determine visible persistence. Two recent studies showed that iconic memory (i.e. visible persistence) decay is not impaired in patients with schizophrenia [55], [56]. The difference between these studies and the one here is that task demands differ strongly. In our temporal integration task the SOA (visible persictence) is directly needed to solve the integration of the two form parts. In the studies mentioned this is not the case. Up to now and to the best of our knowledge, no study has investigated visible persistence with a direct paradigm such as the one used here, adopted from Hogben and DiLollo [41].

There were no differences in asynchrony perception between schizophrenic patients and healthy controls as tested through a temporal figure-ground segmentation task. Hence, our results neither support the notion of a prolonged window of simultaneity [20] nor of a general lower temporal resolution. Could this be due to a floor or seeling effect? This possibility is rather unlikely in the third experiment, because we are measuring directly perceptual thresholds. A floor effect could only be due to the frequency of the monitor during stimulus presentation (150 Hz). The minimal time difference between two consecutive frames is 6.6 ms. Both, patients and controls, performed clearly above this minimal measurable threshold.

There are two main differences in this task compared to the first two experiments. First, stimuli were presented in the periphery of the visual field. But second, the stimulus presentation was 3000 ms with no restrictions for eye-movement. So participants could bring the two possible stimulus-locations to their foveal vision on the one hand. On the other hand this measurement prevents the interaction of temporal overlapping spatial components during this task, which might also explain the intact performance of patients with schizophrenia.

### Limitations

There are limitations of the present study. First, all patients were taking neuroleptic medication. However, previous studies did not find an impact of medication on backward masking [10], [57], [58]. Second, patients and controls were not perfectly matched related to age. However, age was included as a covariate. Moreover, performance in backward masking is stable between the age of 18 and 55 years [59]. Third, schizophrenic patients performed worse than healthy controls in the neurocognitive tasks (d2, LPS3 and MWT) but it is rather unlikely that cognitive deficits affect early visual processing except for attention and moreover, patients were as good as controls in one of our tasks. Fourth, our sample of patients with schizophrenia is biased towards negative symptoms. There was, however, no correlation between psychopathology and performance in the patients group in accordance with previous studies using the shine-through masking paradigm [37]. Because our sample size is too small to we could not analyze if our results are only due to patients with negative symptoms, for which is known that visual information processing is more impaired.

### Conclusions

In summary, our results argue strongly against a generally prolonged visual persistence in patients with schizophrenia. We found that both temporal resolution and temporal integration of schizophrenic patients are decreased in both the masking and the temporal integration paradigm, i.e. patients needed longer time intervals than controls in the masking task while needing shorter ones in the temporal integration task, while asynchrony detection was intact [42]. This is a surprising and encouraging result, indicating that temporal precision is retained in schizophrenia, while both resolution and integration are deteriorated.
